# Psychometric and clinical validation of the fear of childbirth questionnaire in a UK population

**DOI:** 10.1111/aogs.70159

**Published:** 2026-02-17

**Authors:** Kayleigh Sheen, Rebecca Hunter, Claire Lyon, Gillian Houghton, Steven Lane, Jane Wilson, Terri‐Lynn Quigley, Pauline Slade

**Affiliations:** ^1^ School of Social Sciences, College of Health, Science and Society University of the West of England Bristol UK; ^2^ Department of Primary Care and Mental Health, Eleanor Rathbone Building, Bedford Street South University of Liverpool Liverpool UK; ^3^ Liverpool Women's Hospital NHS Foundation Trust Liverpool UK; ^4^ Department of Health Data Science, Institute of Population Health University of Liverpool Liverpool UK; ^5^ Patient and Public Involvement Lead Liverpool UK

**Keywords:** antenatal, fear of childbirth, phobia, pregnancy, psychometrics, tokophobia

## Abstract

**Introduction:**

Severe fear of childbirth (FOC) during pregnancy holds significant implications for maternal mental health, decisions about mode of birth, and potentially infant development. Until recently, there were no tools available to measure FOC within a UK population that assessed the full construct using acceptable phraseology. A new tool, the Fear of Childbirth Questionnaire, has been developed but requires full psychometric evaluation. This study aimed to assess the validity and reliability of the Fear of Childbirth Questionnaire and examine possible threshold scores indicating clinical severity.

**Material and Methods:**

Pregnant women (*N* = 540) completed the Fear of Childbirth Questionnaire online alongside additional measures for current/previous obstetric history, anxiety, and depression to test dimensions of validity (ISRCTN62032021). Most women (*N* = 360) then completed the Structured Clinical Interview for Diagnostic and Statistical Manual of Mental Disorders 5th Edition (Research Version) Module F (phobia) with trained interviewers over the telephone. Sensitivity and specificity were calculated. A subsample (*N* = 61) repeated Fear of Childbirth Questionnaire completion 2 weeks later to inform test–retest reliability. Internal consistency, validity (convergent, discriminant, and criterion‐related), unidimensionality, and factor structure were examined.

**Results:**

Internal consistency was excellent (*α* = 0.89; *ω* = 0.89) and test–retest reliability was good (*r* = 0.87). Strong association with the Fear of Birth Scale (*r* = 0.69) indicated convergent validity. Discriminant validity was indicated via moderate correlations with general measures for anxiety (*r* = *0.40–0.53*), and less so with those of depression *(r* = 0.35). Scores were higher for those with current/previous mental health difficulties, previous birth trauma, those preferring an epidural, and those preferring a planned cesarean section, indicative of criterion‐related validity. An optimal cut‐off value of ≥33 total score and ≥4 current impact score is recommended to initiate further exploration of support/intervention needs (ROC Area under the curve = 0.80, 95% CI 0.75:0.85; sensitivity 71.9, specificity 85.4, positive predictive value 39.0, negative predictive value 95.9).

**Conclusions:**

The Fear of Childbirth Questionnaire is psychometrically robust and can identify FOC at levels commensurate with a clinical phobia. It is the only tool purposefully developed and clinically validated to identify FOC in a UK population.

## Graphical abstract

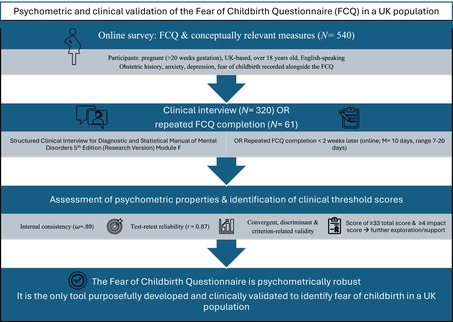

**Abbreviations**
FCQ: Fear of Childbirth Questionnaire
FOBS: Fear of Birth Scale
FOC: fear of childbirth
WDEQ: Wijma Delivery Expectancy/Experience Questionnaire

**Key message**

The Fear of Childbirth Questionnaire is a psychometrically valid tool, which, alongside clinical conversation, can be used to identify fear of childbirth at levels commensurate with a clinical phobia.

## 1 INTRODUCTION

Fourteen percent of pregnant women are estimated to experience severe fear of childbirth (FOC),^1^ resembling a phobia of birth, termed “tokophobia.”^2^ Fear influences decisions about mode of birth, increasing epidural or elective cesarean section requests.^3^, ^4^ Some evidence indicates that heightened antenatal anxiety has been associated with longer labor, increased incidence of preterm birth or birth at a reduced gestational age, and an increased requirement for intervention during labor.^3^, ^5^ Although the association between anxiety and labor duration is not consistently identified.^6^ Sustained stress during pregnancy may hold implications for infant development.^7^ Women with FOC are more likely to experience birth as traumatic, increasing the risk of posttraumatic stress disorder.^8^ Given significant implications for maternal and infant outcomes, methods of accurately identifying FOC are needed.

Currently, the National Institute for Health and Care Excellence (NICE) recommends that pregnant women be routinely asked about general feelings of anxiety and depression;^9^ however, while there can be some comorbidity, FOC is distinctive, and many women will be missed through reliance on nonspecific assessment.^4^, ^10^ Often, specific identification of FOC is reliant on maternal self‐disclosure, and support, if available, is provided by a named midwife, consultant obstetricians, consultant midwives, or perinatal mental health services. Early access to support can be highly beneficial for women,^11^ but this requires effective and accurate identification.

As part of the Five‐Year Forward View Implementation plan, in September 2020, the National Health Service England (NHSE) commissioned new Maternal Mental Health Services (MMHS).^12^ A specific part of their remit is providing FOC intervention, and many services are now introducing routine assessment for FOC. Acceptable, valid, and reliable tools are therefore needed to ensure that services accurately identify those that require intervention, and that care is appropriately targeted.

There is significant heterogeneity in the measurement and inference of the severity of FOC, and limitations in clinical utility.^13^ The most routinely used FOC measure is the Wijma Delivery Expectancy Questionnaire Version A (WDEQ‐A).^14^ Developed in Sweden, items for the WDEQ‐A were constructed based on the authors' clinical experience without input from women themselves. There are issues in item acceptability following the English translation.^15^ Inference of FOC severity is derived from the statistical distribution of scores collected from studies in Sweden,^16^ without comparative testing with UK populations or assessment of clinical relevance. Another measure, the Fear of Birth Scale (FOBS),^17^ has received support as a clinically effective way to open discussions around fears. However, with just two items, the FOBS is insufficient to fully identify the nature of fears.^13^ A newer scale, the Childbirth Fear Questionnaire (CFQ),^18^ has also been developed, with indications of good convergent validity with the WDEQ.^19^, ^20^ However, items on the CFQ were developed by researchers based on existing literature, rather than direct consultation with the target population. Without purposeful assessment of relevance, utility of the CFQ for a UK population is uncertain.

Slade et al. examined acceptability and content validity of several FOC measurement tools with pregnant women both with and without FOC.^15^ The scales examined were the WDEQ‐A, Oxford Worries about Labor Scale (OWLS), Slade–Pais Expectancy of Childbirth Scale and the FOBS.^14^, ^17^, ^21^, ^22^ No measure was both acceptable for women and comprehensive in FOC assessment.^15^ Limitations extended beyond translation and include insufficient content validity. Effective and reliable FOC measurement requires a clearly defined construct.^23^ Slade et al. identified 10 key elements of FOC in women from the UK.^24^ These were the *fear of* (1) *not knowing and not being able to plan for the unpredictable*, (2) *harm or stress to the baby*, (3) *my inability to cope with the pain*, (4) *my body's ability to give birth*, (5) *harm to self in labor and postnatally*, (6) *being “done” to*, (7) *not having a voice in decision making*, (8) *being abandoned and alone*, (9) *internal loss of control* and feeling (10) *terrified of birth and not knowing why*. These key elements were used to create a new measure of fear of childbirth; the Fear of Childbirth Questionnaire (FCQ).

The FCQ was developed for use in clinical practice, grounded in women's own voices and reflecting the full FOC construct.^25^ Slade et al. generated two items for each of the FOC elements, incorporating women's own words.^25^ Three additional items for fear frequency, intensity, and support preferences were included. The FCQ development was closely informed by service user input in relation to clarity and acceptability. The FCQ has a Flesch–Kincaid Grade Level of 5.1 (suitable for new readers), indicating high accessibility. Pilot testing of the FCQ with pregnant women (*N* = 121) was completed, and suggested preliminary indications of internal consistency (*α* = 0.90) and that the items were easy to answer and understand. There is therefore clear evidence that the scale has appropriate content validity and acceptability for UK populations, but a full evaluation of the psychometric properties of the FCQ is required.

The aims of the current study were to (1) evaluate stability of the FCQ via test–retest and internal reliability, and validity via examination of convergent, discriminant, criterion‐related validity, (2) examine the factor structure of the FCQ, and (3) establish clinical thresholds commensurate with clinical severity.

## 2 MATERIAL AND METHODS

### 2.1 Design

A cross‐sectional, online survey with diagnostic telephone interviews and repeated completion of the FCQ was undertaken between June 2023 and May 2024.

### 2.2 Participants

English‐speaking, pregnant individuals under 20 weeks of gestation, over the age of 18, and residing in the United Kingdom were eligible to take part in this study.

### 2.3 Methods

#### 2.3.1 Power calculation

The total sample size (*N* = 420) was informed by the requirements for the test–retest reliability and clinical interview elements of the analysis. The sample size for the test–retest analysis was based on achieving appropriate power to complete a correlation analysis and is aligned with previous research aimed at evaluating test–retest validity of a measurement tool.^26^ A minimum sample size of 40 for test–retest was required. To account for potential attrition, it was intended to invite approximately 60 women to participate in this element of the research. The sample size for clinical interviews was based on an expected prevalence of FOC of 14% (CI 0.12–0.16).^1^ To provide a robust estimate of sensitivity, we aimed to recruit at least 50 women and birthing people who were FOC positive. To achieve this, at the 95% confidence level, 360 participants were recruited. Data was analyzed using SPSS, version 25, Armonk, NY, IBM Corp. A separate power analysis was not performed for objective 2 (examining factor structure of the FCQ), as its sample size requirements were subsumed by those required to examine clinical threshold scores.

#### 2.3.2 Procedure

Ethical approval was obtained from the Southwest National Health Service (NHS) Research Ethics Committee (Reference: 23/SW/0015) and the Health Research Authority (IRAS Reference: 315121). Sponsorship was provided by The University of the West of England. Study advertisements were primarily shared via the host NHS Trust hospital's communication channels (website, social media), or via posters displayed in relevant clinic areas. Advertisements were also shared via the study‐specific social media channels, and via local and national NHS Trusts communication channels. Advertisements included a direct link to the online survey, hosted by Qualtrics.

The online survey incorporated measures for constructs that were conceptually identical (a measure of FOC), distinct from (measures of anxiety, depression), or known to be associated with elevated FOC (mental health history, obstetric history, birth preferences) to facilitate examination of convergent, discriminant, and criterion‐related validity, respectively. A small subsample of participants repeated the FCQ again at least 1 week later (*M* = 10 days, range 7–20 days) to assess test–retest reliability. The remaining participants completed the SCID‐5‐RV over the telephone by a clinical psychologist or research midwife trained in the assessment, within 2 weeks of the online survey. A consultant clinical psychologist within the team provided training and support in relation to the clinical interviews.

#### 2.3.3 Measures

Demographic characteristics (age, ethnicity, town/city of residence, education, employment, prior psychological difficulties) were recorded. The following obstetric history was recorded: parity, mode of any previous births (unassisted vaginal birth, forceps, ventouse, unplanned cesarean section, planned cesarean section, and other), previous birth trauma (yes, no, and prefer not to say), previous pregnancy losses, current antenatal care (midwife‐led, consultant‐led, and unsure), preference for epidural (definitely would like, would consider, definitely do not want, unsure, and prefer not to say), and mode of birth preferences (unassisted vaginal birth, planned cesarean section, unsure, and other).

*The FCQ*.^25^ This is a 20‐item scale measuring ten key elements of FOC as identified by Slade et al.^24^ Items are scored on a scale of 1 (strongly disagree) to 4 (strongly agree) in response to feelings experienced in the previous 2 weeks (e.g., “I am confident I will be able to cope with pain”). Additional items for fear frequency, intensity (1–4), and support preferences are also included. The FCQ yields a total summed score from the 20‐items (range 0–60), and an impact score (frequency × intensity; range 1–16).

To evaluate convergent validity, t*he FOBS*,^17^ measuring fear and worry in relation to birth (2 items), was used. The FOBS is a 2‐item Visual Analog Scale, which asks “How do you feel right now about the approaching birth?” Responses are marked on two 100‐mm lines with anchors “calm/worried” and “no fear/strong fear.” A total score is calculated by averaging the 2 ratings, with a mean of 60+ inferring fear of birth. Internal consistency for the present study was excellent (*α* = 0.93).

Two measures for anxiety were used as constructs that are known to be related to, yet distinct from, FOC. *The State–Trait Anxiety Inventory* (STAI)^27^ records state (transient) and trait (stable) anxiety (40 items). The State Anxiety subscale (20 items) evaluates the current state of anxiety. The Trait Anxiety Scale (20 items) evaluates relatively stable aspects of anxiety, including general states of calmness and security. Items are scored on a scale of 1–4, and a total score is summed (possible range for each subscale: 20–80). Internal consistency for the present study was excellent (State: *ω* = 0.94; Trait: *ω* = 0.93).

The *General Anxiety Disorder 7* (GAD‐7)^28^ measuring generalized anxiety (7 items, scored 0–3) was used. Higher total scores indicate more severe symptoms of anxiety. The GAD‐7 comprises four categories using cut‐off scores to determine clinical severity; 0–4 indicates minimal anxiety symptoms, 5–9 indicates mild anxiety symptoms, 10–14 indicates moderate anxiety symptoms, and greater than 15 indicates severe anxiety symptoms. The internal consistency of the GAD‐7 for the present study was good (*ω* = 0.84).

A measure of depression was incorporated as a construct known to be related to FOC (yet to a lesser extent than anxiety); *the Patient Health Questionnaire 9* (PHQ‐9)^29^ measures depression symptoms (9 items, scored 0–3). Higher scores indicate stronger clinical severity. The PHQ‐9 is used to measure depressive symptoms, with high scores indicating high levels. The PHQ‐9 comprises five categories using cut‐off scores to determine clinical severity; 0–4 indicates no depressive symptoms, 5–9 indicates mild depressive symptoms, 10–14 indicates moderate depressive symptoms, 15–19 indicates moderately‐severe depressive symptoms, and 20–27 indicates severe depressive symptoms.^30^ Internal consistency for the present study was good (*ω* = 0.84).

To identify threshold scores on the FCQ, *the Structured Clinical Interview for DSM‐5 Research Version* (SCID‐5‐RV)^31^ was used to infer diagnoses as specified within the DSM‐5. It is considered to be the “gold standard” examination for diagnosing anxiety disorders. The SCID‐5‐RV was used to diagnose tokophobia, using Module F, Specific Phobia, comprised of: Criterion (A) marked fear or anxiety about a specific object or situation (childbirth), Criterion (C) the phobic situation(s) is actively avoided, or endured with intense fear or anxiety, and Criterion (F) the fear, anxiety, or avoidance causes clinically significant distress or impairment in social, occupational, or other important areas of functioning. Participants were not informed of the SCID‐5‐RV outcome.

### 2.4 Statistical analysis

The demographic and obstetric data were summarized using standard summary statistics. The Shapiro–Wilk test and histograms were used to determine whether continuous data were normally distributed or skewed. Standard hypothesis tests (chi‐square, independent samples *t*‐test, etc.) were used to determine whether there were any between‐group differences between those who completed the FCQ only and those who completed the FCQ and clinical interview. All hypothesis testing was undertaken at the 5% significance level.

The following analyses were completed to assess the psychometric properties of the FCQ.

1. Internal consistency of the FCQ was examined via Cronbach's Alpha and McDonald's Omega coefficients, and confidence intervals were calculated for these coefficients.
2. Test–retest reliability was assessed via correlations between the FCQ completed at time point 1 and repeated completion within 2 weeks of initial completion using the intraclass correlation coefficient.
3. Unidimensionality was assessed via item‐to‐total correlations.
4. Convergent validity was assessed via comparison of FCQ scores with the FOBS using Pearson's correlation coefficient. A strong, positive correlation was expected.
5. Discriminant validity was assessed via comparison of FCQ scores with those obtained from the STAI, GAD‐7, and PHQ‐9 (Pearson's *r*). Moderate/positive associations with GAD‐7/STAI scores and a reduced yet positive association with PHQ‐9 scores were expected.
6. Criterion‐related validity was examined via comparison of subgroups who (i) were currently receiving mental health support compared to those who were not, (ii) had accessed mental health support in the past 5 years compared to those who had not, (iii) reported previous birth trauma compared to those who had not, (iv) had a future preference for epidural compared to those who did not, (v) had previous experience of pregnancy loss compared to those who had not, (vi) had a future preference for elective cesarean section compared to vaginal delivery, and, finally, (vii) those who were receiving consultant‐led care compared to midwifery‐led care. Comparisons between groups were computed (two groups: independent samples *t*‐test, > two groups ANOVA). Post‐hoc group differences were examined using *t*‐tests. Higher FCQ total scores were expected for those who had current/historic support for mental health, reported a previous birth trauma, had a future preference for epidural, had experienced pregnancy loss, had a future preference for cesarean section, and were receiving consultant‐led care.
7. Factor analysis was used to evaluate whether the FCQ items could be grouped into clusters representing different dimensions of the construct under study.^32^

Sensitivity and specificity were calculated for a range of possible cut‐off values indicative of the presence of fear of childbirth. receiver operating characteristic (ROC) curve analysis was used to assess the discrimination properties of the FCQ, and an optimum cut‐off was identified. Kappa value, sensitivity, specificity, positive and negative predictive validity were also determined.

A key methodological element was to assess Inter‐rater reliability for the trained interviewers completing the coding of the SCID. Trained interviewers were not aware of overall FCQ score prior to SCID interview. Their rating (i.e., presence of specific phobia vs. no presence of specific phobia) was assessed using an unweighted Cohen's Kappa during their training for using the SCID‐5‐RV and throughout the study to reduce potential researcher drift.^33^ The research clinical psychologist (RH) double‐coded the first 20 interviews of both research midwives (CL & JLK) and subsequently all the interviews that the RMs screened as positive (tokophobic) and 10% of the interviews that the RM screened as negative (not tokophobic) after a full screen interview. Ten percent of the clinical psychologist's interviews were double‐coded by a second rater. Cohen's kappa statistic indicated good agreement between raters with 96% agreement (k = 0.89, SE = 0.06, 95% CI = 0.77–1.00).

## 3 RESULTS

### 3.1 Participant characteristics

Participants were between 4 and 19 weeks gestation (*M* = 10 weeks), aged on average 32 years (*M* = 31.58, SD = 4.26), mainly of white ethnicity (*N* = 497, 92%), in a relationship (*N* = 497, 93%), employed (*N* = 394, 73%) and with a higher education level (*N* = 441, 82%). Comparisons between those who completed the FCQ only and those who completed the FCQ and clinical interview indicate few differences; specifically, those who completed the FCQ and clinical interview were more likely to be single and employed, and reported a slightly lower fear of childbirth (*N* = 360, *M* = 30.68, SD = 10.37) compared to those who completed the FCQ only (*N* = 119, *M* = 32.91, SD = 10.20) *t*_477,0.05_ = 2.05, *p* = 0.04. See Table 1.

**TABLE 1:** Participant demographics (*N* = 540).

	*N* (%)
Age	
Mean (SD)	31.58 (4.26)
Range	(19, 44)
English as a first language	
No	33 (6.1)
Yes	507 (93.9)
Gender	
Female	540 (100.0)
Ethnicity	
White	497 (92.0)
Mixed	16 (3.0)
Black	8 (1.5)
Asian	18 (3.3)
Prefer not to say	1 (0.2)
Marital status	
Single	43 (8.0)
In a Relationship	497 (92.8)
Education	
Secondary School	99 (18.3)
University	441 (81.7)
Employment	
Employed	394 (73.0)
Not Employed	146 (27.0)

Abbreviation: SD, standard deviation.

Just over 5 percent (*N* = 30, 5.5%) reported scores indicative of moderate to severe depression, and 2 percent (*N* = 13, 2.4%) reported severe depression. For general anxiety, just under 10 percent (*N* = 53, 9.8%) reported moderate to severe anxiety, and 7 percent (*N* = 42, 7.7%) reported severe levels of anxiety. According to scores reported on the FOBS, just under 40 percent (*N* = 206, 38%) were reporting severe levels of FOC. There was no correlation between gestational age and level of fear of childbirth as reported by the FCQ (*r* = 0.05).

### 3.2 Reliability

#### 3.2.1 Internal consistency

Overall, the FCQ demonstrated good internal consistency (*α* = 0.89; 95% CI 0.876: 0.904; *ω* = 0.89; 95% CI 0.869: 0.901).

#### 3.2.2 Test–retest reliability

There was a strong positive correlation between FCQ completion at time points 1 and 2 (*r* = 0.87), indicating good test–retest reliability.

### 3.3 Validity

#### 3.3.1 Unidimensionality

Unidimensionality was assessed via item‐to‐total correlations (Table S1). All items were positively correlated; therefore, no items were discarded.

#### 3.3.2 Convergent and discriminant validity

There was a strong positive correlation between the FCQ and FOBS (*r* = 0.69), indicative of convergent validity.

As expected, there was a moderate positive correlation between the FCQ total score and GAD‐7 (*r* = 0.40), and a strong positive correlation between FCQ Total and STAI (State *r* = 0.53, Trait r = 0.52). In addition, results indicate a moderate positive correlation between FCQ Total and PHQ‐9 (*r* = 0.35), suggesting adequate discriminant validity. Correlation coefficients are reported in Table 2.

**TABLE 2:** Means, standard deviations, and correlations between FCQ total and other mental health measures.

Variable	*N*	*M*	SD	1	2	3	4	5
1. FCQ total	540	31.35	10.38	—				
2. PHQ‐9 total	540	6.59	4.86	0.352	—			
3. GAD‐7 total	540	5.28	5.13	0.398	0.709	—		
4. STAI state total	540	43.01	12.04	0.538	0.619	0.755	—	
5. STAI trait total	540	42.38	11.73	0.521	0.651	0.738	0.886	—
6. FOBS total	537	51.35	26.32	0.687	0.261	0.388	0.515	0.452

Abbreviations: *M*, mean; SD, standard deviation.

#### 3.3.3 Criterion‐related validity

Results indicated significantly higher FCQ Total scores in those who were receiving support for their mental health (*M* = 35.00, SD = 8.65), those who had received support in the previous 5 years for their mental health (*M* = 33.40, SD = 10.34), those who had experienced a previous birth as traumatic (*M* = 30.76, SD = 9.35), those with a preference for epidural (*M* = 33.85, SD = 9.75), and those who had cesarean section as their preferred mode of birth (*M* = 34.42, SD = 10.66). There were no significant differences in FCQ Total scores between those who had experienced pregnancy loss (*M* = 30.25, SD = 10.27) and those who had not (*M* = 28.42, SD = 10.32). Table 3 shows the criterion‐related validity comparisons.

**TABLE 3:** Criterion‐related validity comparisons.

	No	Yes	*T*‐test
Current support for mental health difficulties			
*M* (SD) FCQ total	30.80 (10.51)	35.00 (8.65)	*p* < 0.001**
Range	(3, 60)	(14, 52)	
Mean difference	4.20		
95% CI	(1. 62, 6.77)		
Previous support for mental health difficulties			
*M* (*SD*) FCQ total	29.14 (9.98)	33.40 (10.34)	*p* < 0.001**
Range	(3, 55)	(4, 60)	
Mean difference	4.26		
95% CI	(2.54, 5.98)		
Previous pregnancy loss *N* = 258			
*M* (SD) FCQ total	28.42 (10.32)	30.25 (10.27)	*p* = 0.078
Range	(3, 49)	(3, 57)	
Mean difference	1.83		
95% CI	(‐0.70, 4.36)		
Previous birth perceived as traumatic *N* = 211			
*M* (SD) FCQ total	22.05 (9.06)	30.76 (9.35)	*p* < 0.001**
Range	(4, 40)	(3, 57)	
Mean difference	8.71		
95% CI	(5.90, 11.51)		
Preference for epidural in future birth			
*M* (SD) FCQ total	30.76 (10.45)	33.85 (9.75)	*p* = 0.003*
Range	(3, 56)	(4, 60)	
Mean difference	3.09		
95% CI	(0.88, 5.30)		

	Vaginal	Elective CS	*T*‐test
Mode of birth preference for future birth			
*M* (SD) FCQ total	30.87 (10.26)	34.42 (10.66)	*p* = 0.003*
Range	(3, 57)	(3, 60)	
Mean difference	3.55		
95% CI	(1.02, 6.09)		

Abbreviations: CI, confidence interval; *M*, mean; SD, standard deviation.

* *p* significant at the 0.05 value.

** *p* significant at the <0.001 value.

It was hypothesized that fear of childbirth would be higher in those receiving consultant‐led care. Results indicated a significant difference between the groups, but also several participants who were unsure which form of care they were receiving. Following advice from the study's PPI advisors, comparisons were undertaken between those receiving midwife‐led care and those unsure/receiving consultant‐led care. Post‐hoc comparisons indicate a significant difference between those receiving midwife‐led care (*M* = 30.47, SD = 10.55) and those who were not sure or who reported consultant‐led care (*M* = 33.55, SD = 9.28). All other comparisons were non‐significant.

### 3.4 Exploratory factor analysis

Exploratory factor analysis (EFA) was used to identify underlying dimensions. EFA was used to determine the structure of the data and the number of factors needed to explain the relationships between the items. The KMO index and Bartlett's test of sphericity indicated that the data were suitable for factor analysis (KMO = 0.90, Bartlett's test *p* < 0.01). Factors were extracted from the correlation matrix using principal component analysis (PCA). A varimax rotation was then applied to the factors to group loadings either close to zero or one. Factors with an eigenvalue >1 were retained in the model. Where there were similar loadings onto two separate factors, allocation to a factor was based on clinical coherence following team discussion. Results indicate that items load onto four factors (overall 57% variance explained), which, following PPI input, are referred to as: (1) confidence in self (six items, 33.4% variance explained; *ω* = *0*.84; 95% CI 0.820: 0.864), (2) healthcare professionals providing support (five items, 11.5% variance explained; *ω* = *0*.82; 95% CI 0.791: 0.845), (3) harm to self and baby (seven items, 7.1% variance explained; *ω* = *0*.75, 95% CI 0.71: 0.790), and (4) birth as a threat (two items, 5.0% variance explained; *α* = *0*.50; 95% CI 0.399: 0.579). Table 5 shows the factor loadings.

#### 3.4.1 Examination of clinical thresholds

**FIGURE 1:**
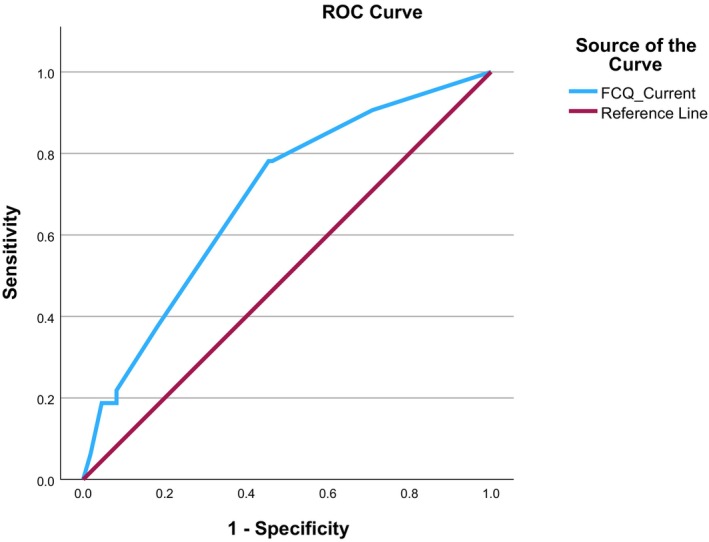
ROC curve analysis for FCQ current impact. FCQ, Fear of Childbirth Questionnaire; ROC, Receiver Operating Characteristic.

The prevalence of FOC as indicated by the SCID‐5‐RV was 11% (*N* = 41/360, 11.39%). ROC curve analysis was used to assess the discrimination properties of the FCQ total score and FCQ current impact score. For the FCQ total score, ROC analysis provided an area under the curve = 0.78 (95% confidence interval [0.71, 0.85]; Figure S1). The optimal cut‐off value with respect to optimizing sensitivity and specificity was 34.5 (Figure S2). Considering the current impact score, ROC analysis indicated an area under the curve = 0.80 (95% confidence interval [0.75, 0.85]; Figure 1). The optimum cut‐off for Current Impact is 4. However, in both cases, it is often desirable to use a slightly lower score, which increases sensitivity for a loss in specificity and ensures more potentially positive cases are identified (Figure S3). Table 4 provides the summary statistics for a range of cut‐off values and combinations of the two scores.

**TABLE 4:** Sensitivity and specificity values by FCQ total/current impact cut‐off score.

Cut‐off score	Sensitivity	Specificity	PPV	NPV
FCQ total 34.5	75.6	67.4	23.0	95.6
FCQ total 32.5	82.9	62.0	21.9	96.6
FCQ total 30.5	87.8	53.0	19.4	97.1
FCQ total 32.5 and CI >3	71.9	85.4	39.0	95.9
FCQ total 32.5 or CI >3	56.1	88.7	39.0	94.0

Abbreviations: CI, current impact; NPV, negative predictive value; PPV, positive predictive value.

**TABLE 5:** Exploratory factor analysis loadings FCQ item.

	Factor loading			
	1	2	3	4
Factor 1 (“confidence in self”)				
1. I feel fine about my labor and giving birth to my baby	0.69	0.12	0.30	−0.03
5. I am confident I will be able to cope with the pain	0.74	0.01	0.16	0.18
7. I worry I will lose control of myself during labor	0.50	0.08	0.23	0.44
8. I am confident my body can give birth to my baby	0.76	0.15	0.13	0.08
10. I am confident I am emotionally strong enough to cope with labor and birth	0.66	0.15	0.16	0.32
20. I am confident my body will work well during labor and birth	0.78	0.13	0.23	0.07
Factor 2 (“healthcare professionals providing support”)				
3. I am confident that staff will always respect my wishes	0.00	0.85	0.04	0.01
9. I worry I will not have a voice in decision‐making during labor	0.03	0.76	0.22	0.19
12. I am worried about things being “done” to me during labor and birth	0.03	0.63	0.42	0.10
14. I am confident that staff will be there when I need them	0.19	0.76	0.05	0.06
17. I am confident I will get the pain relief I want	0.42	0.59	0.07	0.10
Factor 3 (“harm to self and baby”)				
2. I worry my labor or birth will not go to plan	0.26	0.16	0.63	−0.03
4. I am worried about the long‐term effects that labor or birth could have on my body	0.23	0.10	0.43	0.09
6. I am worried that my baby will be harmed during labor and birth	0.09	0.12	0.72	0.14
11. I worry that labor is unpredictable	0.41	−0.01	0.59	0.04
13. I am worried I will be harmed during labor	0.18	0.52	0.52	0.02
15. I worry that my baby will feel distressed during labor and birth	0.05	0.06	0.69	0.29
16. I worry about having unpleasant procedures during labour and birth	0.26	0.28	0.58	0.10
Factor 4 (“birth as a threat”)				
18. I worry about being left alone, without my chosen birth partner, during labor	0.07	0.33	0.11	0.73
19. I am worried about labor and birth and I don't know why	0.34	−0.03	0.21	0.67

## 4 DISCUSSION

Findings indicate that the FCQ is a reliable and valid measurement tool, which can accurately identify clinically relevant levels of fear of childbirth early in pregnancy. This is the first tool specifically developed to (1) measure FOC within a UK population and (2) that has been validated using a clinician‐administered diagnostic interview. Previous studies in Italy and Canada have conducted clinical diagnostic interviews alongside the Wijma Delivery Expectancy/Experience Questionnaire (WDEQ)^34^ or new items developed for a Canadian population,^18^ but neither tool provides a comprehensive assessment of fear for a UK population. The FCQ provides a valid and reliable method of identifying women experiencing clinically relevant FOC, and for whom further support/intervention may be beneficial.

The prevalence of clinically relevant levels of FOC as reported by the present study was 11%. This is similar to, albeit slightly lower than, global estimations of 14%,^1^ and commensurate with the prevalence of FOC reported across six European countries,^35^ neither of which incorporated the use of clinical interviews. The prevalence of FOC reported in this present study is higher than other UK‐based estimations. For example, secondary analysis of data from 545 women who completed a clinical interview at 28 weeks' gestation reported a population prevalence estimate for specific phobia of 8.4% (95% CI: 5.8–12.1%), and a prevalence estimate of tokophobia of 0.032% (95% CI: 0.0044–0.23%).^10^ Arguably, online research is likely to include an element of selection bias; however, further research using either clinical diagnostic interviews or tools with clinically validated thresholds is required.

Interestingly, the level of FOC reported by participants did not differ by gestational age. It is sometimes proposed that levels of fear fluctuate throughout pregnancy, rising during the third trimester;^36^ however, longitudinal research has challenged this assumption. Using the FOBS at 17–19 weeks' gestation and 32–34 weeks' gestation, Hildingsson et al. reported an overall prevalence of 22% in mid pregnancy and 19% in late pregnancy;^37^ a statistically significant decrease over time. Further examination of the trajectory of FOC throughout pregnancy is warranted.

This study is one of the few, especially in the United Kingdom, that have employed a clinical interview to determine the presence of FOC. Inter‐rater reliability for the SCID‐RV‐5 was indicative of good agreement between the trained interviewers. Completion of the clinical interview provided a basis upon which to confirm participant eligibility; however, it is noted that for a small minority of participants, and as with the nature of online research, eligibility was reliant on self‐report. Further evaluation of unidimensionality and instrument structure via confirmatory factor analysis (CFA) is required. The authors have plans to complete a CFA on another sample, which will be the subject of a separate paper. In stage one, women were aged on average 31.6 years, similar to national statistics (30.9 years).^38^ A higher proportion of women were educated at the university level (81.7%) compared to national proportions. This may in part be due to online recruitment, whereby individuals who are more highly educated may be more likely to participate in online research. Most participants were of white ethnicity (92.0%), with English as their first language (93.9%), highlighting a requirement to enhance representation of individuals from ethnic minority groups. Future research should look to gain a more representative sample of pregnant women.

The FCQ is a valid, reliable tool that can be used to identify FOC commensurate with a clinical diagnosis of phobia. It is the first clinically validated tool for FOC that has been purposefully developed for use with a UK population. Continued application of tools to measure FOC requires recognition that the content of fear will vary between countries. Recognition of cultural sensitivity and item phraseology following translation is fundamental for accurate and comprehensive measurement. It may be, for example, that new items are required (or items need to be removed).

Recently, a systematic review of FOC tools recommended use of the FCQ, based primarily on construct validity.^39^ A recent investigation into the feasibility and acceptability of a brief Acceptance and Commitment Therapy intervention for women experiencing FOC identified a reduction of fear as measured by the FCQ and the FOBS over time,^40^ indicative of potential utility of the FCQ for change evaluation. Further exploration is required.

## 5 CONCLUSION

The FCQ is now a fully tested, valid, and reliable tool available with recommended clinical cut‐off values to be used in conjunction with clinical conversation. The provision of targeted support for FOC is a high priority currently within the National Health Service's Maternal Mental Health Services. This necessitates accurate identification at a time early enough in pregnancy to facilitate meaningful psychological intervention. The FCQ can provide a means of initiating discussions about FOC with clinical care providers, facilitating the identification of those for whom further support may be beneficial.

## AUTHOR CONTRIBUTIONS

Conceptualization, KS, PS, GH, SL; methodology, KS, PS, GH, SL, TQ, JW; validation, SL; formal analysis, SL; investigation, RH, CL, JW; resources, RH, CL, KS, PS, GH, JW, SL, TQ; data curation, RH, CL, SL; writing—original draft preparation, KS, RH; writing—review and editing, KS, RH, CL, GH, JW, TQ, SL, PS; visualization, SL, RH, KS; supervision, KS, PS, RH; project administration, RH, KS, PS; funding acquisition, KS, PS, GH, SL, TQ. All authors have read and agreed to the published version of the manuscript.

## FUNDING INFORMATION

This project is funded by the National Institute for Health and Care Research (NIHR) under its Research for Patient Benefit (RfPB) Programme (Grant Reference Number NIHR203154). The views expressed are those of the author(s) and not necessarily those of the NIHR or the Department of Health and Social Care.

## CONFLICT OF INTEREST STATEMENT

All authors report no conflict of interest.

## ETHICS STATEMENT

Ethical approval was obtained from the Southwest National Health Service (NHS) Research Ethics Committee (Reference: 23/SW/0015) on March 14, 2023 and the Health Research Authority (IRAS Reference: 315121) on March 14, 2023. Sponsorship was provided by the University of the West of England, Bristol.

## Supporting information


**Figure S1.** ROC curve analysis for FCQ total score.
**Figure S2.** Sensitivity and specificity for FCQ total score.
**Figure S3.** Sensitivity and specificity for FCQ current impact.

**Table S1.** Item‐to‐total‐minus‐item Correlations.

## Data Availability

Research data are not shared.
