# Supplementary material for: Psychometric and clinical validation of the fear of childbirth questionnaire in a UK population
Source: Acta Obstet Gynecol Scand. 2026 Feb 17;105(4):748–57. doi: 10.1111/aogs.70159 (PMC13140680; doi:10.1111/aogs.70159)
Supplement: Supplementary file 1 — Figure S1. ROC curve analysis for FCQ total score. Figure S2. Sensitivity and specificity for FCQ total score. Figure S3. Sensitivity and specificity for FCQ current impact. [file AOGS-105-748-s002.docx]

SUPPLEMENTARY INFORMATION


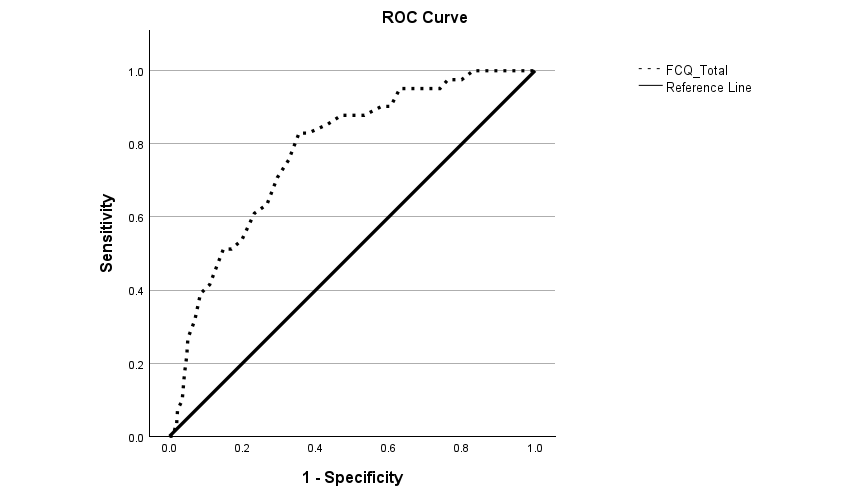


**FIGURE S1.** *ROC Curve Analysis for FCQ Total Score*


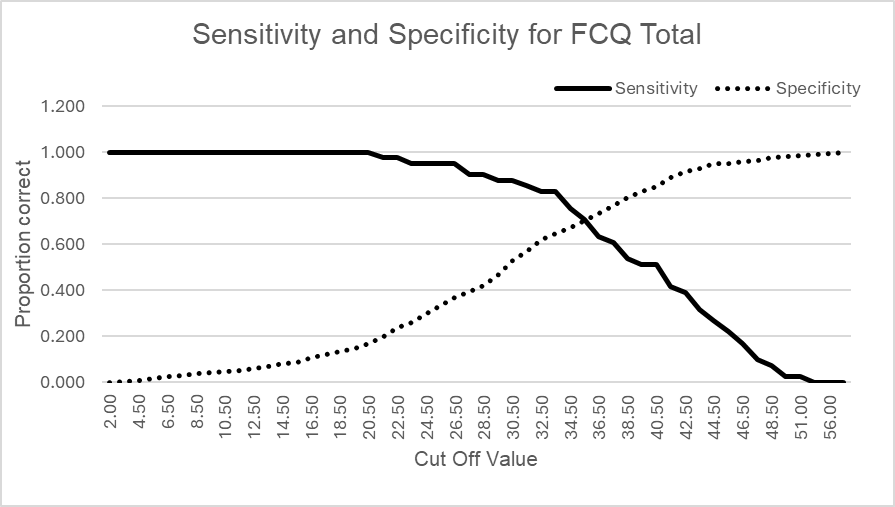


**FIGURE S2:** *Sensitivity and Specificity for FCQ Total Score*


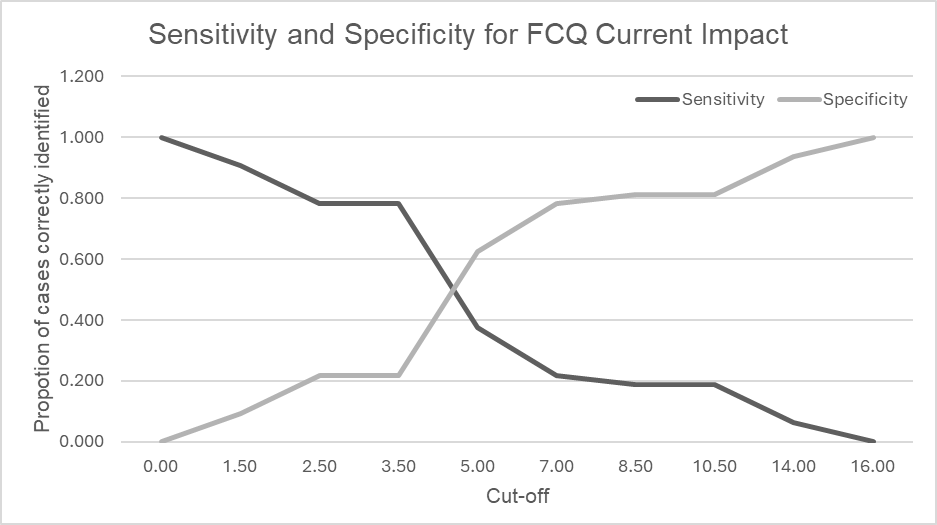


**FIGURE S3***: Sensitivity and Specificity for FCQ Current Impact*
