# Supplementary material for: Psychometric and clinical validation of the fear of childbirth questionnaire in a UK population
Source: Acta Obstet Gynecol Scand. 2026 Feb 17;105(4):748–57. doi: 10.1111/aogs.70159 (PMC13140680; doi:10.1111/aogs.70159)
Supplement: Supplementary file 2 — Table S1. Item‐to‐total‐minus‐item Correlations. [file AOGS-105-748-s001.docx]

| **Table S1***: Item-to-total-minus-item Correlations* | |
| --- | --- |
| FCQ Item | *R* |
| 1. I feel fine about my labour and giving birth to my baby | 0.530 |
| 2. I worry my labour or birth will not go to plan | 0.509 |
| 3. I am confident that staff will always respect my wishes | 0.393 |
| 4. I am worried about the long-term effects that labour or birth could have on my body | 0.397 |
| 5. I am confident I will be able to cope with the pain | 0.510 |
| 6. I am worried that my baby will be harmed during labour and birth | 0.490 |
| 7. I worry I will lose control of myself during labour | 0.530 |
| 8. I am confident my body can give birth to my baby | 0.551 |
| 9. I worry I will not have a voice in decision making during labour | 0.523 |
| 10. I am confident I am emotionally strong enough to cope with labour and birth | 0.586 |
| 11. I worry that labour is unpredictable | 0.510 |
| 12. I am worried about things being ‘done’ to me during labour and birth | 0.532 |
| 13. I am worried I will be harmed during labour | 0.607 |
| 14. I am confident that staff will be there when I need them | 0.477 |
| 15. I worry that my baby will feel distressed during labour and birth | 0.466 |
| 16. I worry about having unpleasant procedures during labour and birth | 0.587 |
| 17. I am confident I will get the pain relief I want | 0.549 |
| 18. I worry about being left alone, without my chosen birth partner, during labour | 0.424 |
| 19. I am worried about labour and birth and I don’t know why | 0.439 |
| 20. I am confident my body will work well during labour and birth | 0.604 |
